# Autoimmune diseases affecting skin melanocytes in dogs, cats and horses: vitiligo and the uveodermatological syndrome: a comprehensive review

**DOI:** 10.1186/s12917-019-2003-9

**Published:** 2019-07-19

**Authors:** Heng L. Tham, Keith E. Linder, Thierry Olivry

**Affiliations:** 1 Department of Small Animal Clinical Sciences, Virginia-Maryland College of Veterinary Medicine, Virginia Tech, Blacksburg, VA USA; 2 Comparative Medicine Institute, College of Veterinary Medicine, North Carolina State University, Raleigh, NC USA; 3 Department of Population Health and Pathobiology, College of Veterinary Medicine, North Carolina State University, Raleigh, NC USA; 4 Department of Clinical Sciences, College of Veterinary Medicine, North Carolina State University, Raleigh, NC USA

**Keywords:** Autoimmune skin diseases, Vitiligo, Uveodermatological, Vogt-Koyanagi-Harada, Leukoderma, Depigmentation, Melanocytes, Canine, Feline, Equine, Dermatology

## Abstract

Autoimmune dermatoses targeting melanocytes have gained attention in human medicine due to their progressive nature and the social impact suffered by affected individuals. In veterinary medicine, vitiligo and the uveodermatological syndrome are the two autoimmune diseases that are known to affect skin melanocytes.

In the first part of this article, we will review the signalment, clinical signs, histopathology and the treatment outcome of vitiligo in dogs, cats and horses; where pertinent, we compare the animal diseases to their human homologue. In a similar fashion, the information on the uveodermatological syndrome in dogs is reviewed and, where relevant, it is compared to the Vogt-Koyanagi-Harada (VKH) syndrome in humans.

Canine, feline and equine vitiligo have many features that mirror their human counterparts. The most effective treatment and outcome of vitiligo in animals remain unclear. The canine uveodermatological syndrome resembles the incomplete VKH variant in humans; for affected individuals, an immediate diagnosis and aggressive treatment are crucial to prevent the development of blindness.

**Electronic supplementary material:**

The online version of this article (10.1186/s12917-019-2003-9) contains supplementary material, which is available to authorized users.

## Background

Melanocytes possess the unique ability to synthesize melanin pigments, which contribute to the skin and hair color of humans and animals. Other than the skin and skin appendages of domesticated mammals, melanocytes are found in the oral mucosa, eye, cochlea and less consistently the meninges [1]. In the eyes, melanocytes are abundant in the uvea (i.e., the iris, ciliary body and choroid) [1]; the uveal pigment is thought to protect the retina from an overexposure to solar radiation and its resulting damaging affects [2]. In the ear, melanocytes are found in the *stria vascularis* of the cochlea and contribute to endolymph formation [1].

Autoimmune diseases of melanocytes have gained attention in human medicine due to their often-progressive nature and the negative social impact suffered by affected individuals. These diseases are due to the specific destruction of melanocytes, which results in a variety of clinical presentations, depending on the organ(s) in which the melanocytes are targeted. In vitiligo, signs associated with the destruction of melanocytes are limited to the skin, lips and/or oral cavity. In contrast, in the Vogt-Koyanagi-Harada (VKH) syndrome, they are more diverse because of involvement of the skin, lips, oral cavity, eyes, meninges and/or auditory system.

In this review, we will focus on the two recognized autoimmune disease of melanocytes in animals: vitiligo and the uveodermatological syndrome, the canine homologue of the VKH disease of humans.

### Vitiligo

#### Introduction

Vitiligo is defined as an acquired, chronic, depigmentation disorder characterized by white patches, often symmetrical in humans, corresponding to a substantial loss of functional epidermal and sometimes hair follicle melanocytes [3]. The term vitiligo was first used in the Latin medical classic ‘*De Medicina’* during the second century (cited in [4]). There are different opinions on the origin of the word vitiligo: while some state that the depigmentation resembles the white glistening of the flesh of calves (in Latin, calf is ‘*vitulus’* with ‘*vituli’* its plural), others believed that the term vitiligo is derived from the Latin word ‘*vitium’*, meaning defect or blemish [5].

Herein, we review the relevant information published to date on the canine, feline and equine vitiligo and we compare it with that published on the human disease homologue, where appropriate.

#### Historical perspective

Vitiligo in humans was first described more than 1500 years BC [6]. In ancient times, vitiligo was confused with leprosy and other depigmenting disorders [6], and it resulted in a discrimination and social stigma that, unfortunately, still persists today [4]. It is only in the last century that vitiligo has gained more interest in the field of medical research [6].

The first case series of vitiligo in dogs was reported in 1971 [7]. Seven years later, two articles were published: one as a case report in the proceedings from a human dermatology meeting in Geneva [8], and the other as a prospective study that collected data via a questionnaire and reported three clinical cases [9]; the latter article was the first to detail the clinical, histological and electron microscopic features of vitiligo in 38 dogs.

Reports on feline vitiligo are even rarer than those of the canine disease: the first published case reports of feline vitiligo were both in 1986 [10, 11], although this disease was likely recognized earlier. The most detailed report on feline vitiligo (in a Siamese cat) was published in 1994 [12].

In the horse, Duerst was cited to have first mentioned the term vitiligo in 1931 (cited in [13]), but it was not until the 1960s when Meijer, a Dutch veterinarian, reported equine vitiligo in three separate papers [13–15]; of note is that the horses in the 1965 report [13] might be the same as those included in the papers published in 1961 [14] and 1962 [15].

#### Incidence and prevalence

Vitiligo is the most common depigmenting disorder in humans [4], with a prevalence estimated at approximately 0.5–2.0% of the world population [16]; it has been reported to be as high as 8.8% in India [17]. The disparity between prevalence and incidence could be due to the demographics from which the data originated (e.g., it might be estimated to be higher in dark skinned populations in whom lesions are more prominent) [4].

There are no available data to estimate the global or regional incidence and prevalence of vitiligo in dogs, cats or horses. However, at Cornell University, Scott and Miller reported that vitiligo accounted for 0.7% of equine dermatoses examined at the veterinary teaching hospital [18]. The prevalence of vitiligo in animals may be much higher than that reported, however, because this disease is a primarily cosmetic issue in animals, and it might not motivate owners to seek any veterinary care.

#### Etiopathogenesis

Most of the information available about the etiopathogenesis of vitiligo derives from results of studies performed using human samples. The three main hypotheses are biochemical, neural and autoimmune [19]. Other review papers suggested the adhesion defect [17] and oxidative theories [19], even though the latter overlaps with the biochemical hypothesis. Of these theories, the autoimmune hypothesis is the leading one [17], as it is derived from studies that showed that antibodies directed against melanocytes were more prevalent in animals and humans with vitiligo [19]. Other studies also showed the involvement of the innate immune system (natural killer cells and inflammatory dendritic cells) and cytotoxic CD8^+^ T lymphocytes [16]. This explains why most interventions for the treatment of vitiligo in humans are centered around immunosuppressive therapies. In a recent review, the convergence theory posits that a combination of various pathways is involved in the development and progression of vitiligo [19]. These mechanisms, in addition to the aforementioned, included genetic susceptibility, mechanical stress and associated Koebnerization (i.e., trauma-induced lesions), psychological stress, reduced melanocytes antioxidant defenses, microbial dysbiosis and aberrant melanocytes-keratinocyte intercellular communication, which all intertwined in a yet-unknown mechanism leading to the loss of melanocytes from the skin. A recent theory attempts to explain this convergence mechanistically [20]: an oxidative cellular stress would not only injure melanocytes but also induce novel autoantigens, or expose cryptic cellular antigens, which would then activate an immune response against melanocytes to promote lesion development. Additional factors, (e.g., genetic, environmental, etc.) could then influence both mechanisms of oxidative injury and immunological responses, thus explaining the multifactorial nature of the disease.

In 1986, Naughton and colleagues [10] reported the detection of antibodies to surface antigens of pigmented cells in 24 animals with vitiligo. In that study, serum was collected from 28 Belgian Tervueren (17 with vitiligo), seven Siamese cat (four with vitiligo) and six Arabian horses (three with vitiligo). Using immunoprecipitation and immunofluorescence assays and human pigmented melanoma cells previously shown to express vitiligo antigens, all animals with vitiligo had circulating antibodies to a surface antigen of pigmented cells, whereas none of those from normal animals had such antibodies. The authors suggested that the pathogenesis of vitiligo in man and animals was similar because both appeared to have a similar abnormal antibody response to pigmented cells. However, since then, to the authors’ knowledge, there have been no new studies on the pathogenesis of vitiligo in animals, thus limiting the advancement of mechanistic-based treatment and outcome assessment.

#### Signalment

In humans, vitiligo affects both genders equally [16]. It can develop at any age, but 70–80% of cases arise before the age of 30 with an onset before the age of 12 years being common (up to 37% of patients in one study) [17].

In the dog, rottweiler and doberman pinscher dogs (in the USA) as well as collies are thought to be predisposed to vitiligo [11, 21]. In the present review, the breeds reported with canine vitiligo are derived from 12 publications including 74 dogs [7–9, 11, 22–29]. Among these cases, there were 38 Belgian Tervuerens (51%), 15 rottweilers (20%), seven Labrador retrievers (9%), three German shepherd dogs (4%), two old English sheepdogs (3%) and Beauceron shepherd dogs (3%) and one each (1%) of the following breeds: giant schnauzer, miniature dachshund, Newfoundland, Bernese mountain dog, Collie and a mixed breed dog. From the nine case reports in which information on the sex was available [7, 9, 11, 22, 23, 25–29], the female-to-male ratio was 1.0. The mean and median ages of onset were 26 months and 24 months, respectively (range: 2 months to 11 years).

There are four reports of feline vitiligo including seven cats [10–12, 30]. Of these, six were Siamese [10–12], and one was a European mixed breed [30]. The sex was only reported for three cats: two females [11, 12] and one male [30]. The age of onset was only reported in one Siamese, and it was 21 months [12].

The breeds of horses affected with vitiligo can be inferred from five reports including 32 horses [10, 13, 31–33]. Among these cases, there were 12 Gelderlands (38%), nine Spanish thoroughbreds (28%), four Arabians (13%) and Belgians (13%) and one each (3%) of the following breeds: Oldenburg, Mecklenburg and quarter horse. The breeds that were reported by Meijer in two reports published in 1961 and 1962 [14, 15] were not considered because we are unsure if the reported horses were the same reported in the 1965 [13] paper. The sex of the affected horses was available from three reports with 28 horses [13, 32, 33] of which the female-to-male ratio was 2.1. Vitiligo is reported to be common in Arabian horses [18] (the so-called “Arabian fading syndrome” and “pinky Arab”) and there are numerous references listing this breed in review papers, equine text books and the lay literature. The published information is however limited and thus it may not completely represent the commonality of equine breeds affected by vitiligo, which are different in the literature and appear to vary by geographical location. The age of onset was only reported in seven horses [14, 15, 33] with a median of 48 months (range: 1 to 18 years).

#### Clinical signs

In humans, vitiligo is classified clinically into segmental (SV) and non-segmental variants (NSV), with the latter including three major subsets: generalized, acrofacial and universal vitiligos [17]. Other variants are mixed and unclassified vitiligo (focal and mucosal vitiligo) [17]. The initial lesions of NSV in humans usually arise on areas exposed to chronic traumas, especially on the hands or the arms [17], a feature attributed to the Koebner phenomenon [4]. According to a recent international consensus conference [34], NSV is characterized by depigmented macules of varying sizes, which usually spare the scalp and haired regions, although hair depigmentation may occur with disease progression. The loss of hair pigmentation can follow the depigmentation of the skin but rarely precedes it [19]; there is one case series of follicular vitiligo described in humans [35]. Depigmentation of the iris and retina are seen in a minority of patients with vitiligo (reviewed in [17]).

**Fig. 1:**
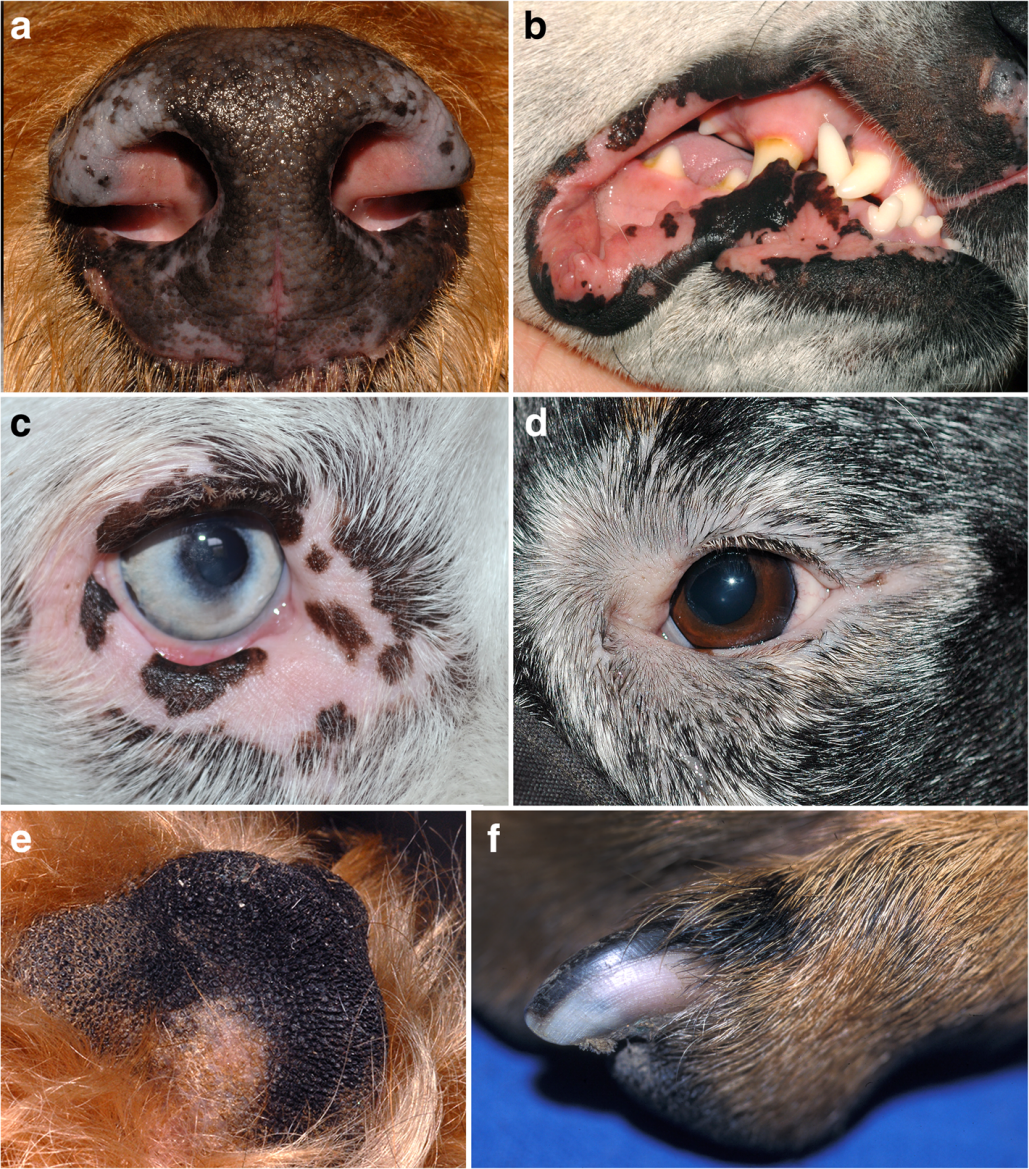
Canine vitiligo. **a** 5-year-old Airedale terrier with a 2-month history of fairly-symmetrical depigmented patches on the nasal planum (courtesy of Dr. F. Banovic, case material NCSU). **b** 4-year-old Australian shepherd with a 3-month history of rapidly-progressing depigmentation (leukoderma) of the nasal planum and lips; hair depigmentation (leukotrichia) was also present on the head (courtesy of Dr. HL Tham, case material NCSU). **c** 4-year-old Bernese Mountain dog with periocular depigmentation; this dog also had leukotrichia that progressed to generalized poliosis (courtesy of Dr. L. Beco). **d** 2-year-old Rottweiler with periocular leukoderma and leukotrichia (courtesy of Dr. E. Guaguère). **e** same dog as in (**a**); depigmentation of the footpad (courtesy of Dr. F. Banovic, case material NCSU). **f** same dogs as in (**d**): partial depigmentation of the claw (courtesy of Dr. E. Guaguère)

**Fig. 2:**
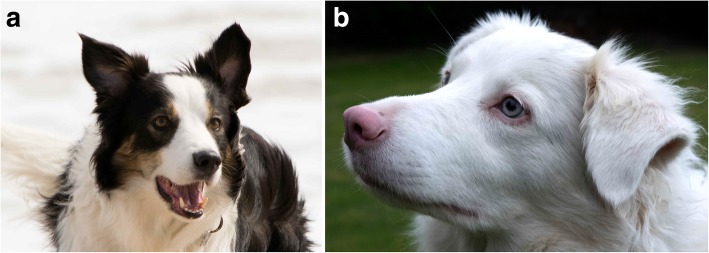
Canine vitiligo. **a** Australian shepherd with a normal coat color. **b** Eight months later, there was generalized skin, hair and iris depigmentation due to vitiligo; the owner confirmed the iris depigmenting as the coat color changed (courtesy of Dr. K. Pantenburg)

In dogs, information on the location of the first lesion(s) of vitiligo was available from seven reports [9, 22–25, 28, 29] including 55 patients. In most dogs, depigmented macules and/or patches initially developed on the face and were more often multifocal than focal. The gingiva and lips were the two most commonly affected regions and the depigmentation sometimes progressed from multifocal to complete oral depigmentation. As the disease progressed, depigmented lesions were confined to the face and/or head in most dogs and involved one or more of the following regions: eyelids, eyelashes, nasal planum, oral cavity (hard palate and buccal mucosa), pinnae and muzzle (Fig. 1). However, the depigmentation also developed at other locations such as the footpads [8, 9, 25, 29], scrotum [9], nails/claws [23, 25], paws/limbs [23–25] and the neck/trunk/rump region [11, 23, 24]. Generalized depigmentation (Fig. 2) was reported in two dogs [27, 28], with the former case only affecting black hair. Bilaterally symmetrical lesions were only reported in three dogs in one case report [22].

The rare form of follicular vitiligo resembles the case reported by White and Batch [24], where all seven Labrador retriever puppies only exhibited leukotrichia without leukoderma.

Interestingly, Mahaffey and colleagues reported that four dogs had focal depigmentation of the outer surface of the lower lip at the point contacted by the maxillary canine tooth [9]. Whether this clinical feature was associated with a Koebner phenomenon or trauma-induced hypopigmentation is not known but the adhesion defect theory (“melanocytorrhagy theory”) has been postulated as one of the pathogenesis of non-segmental vitiligo in humans [17]. Based on the clinical distribution of lesions in dogs, most cases of canine vitiligo resemble those of the acrofacial subset of NSV, which is the most common form of vitiligo in humans, at least during the initial course of the disease [4].

Human vitiligo has been associated with autoimmune thyroid disease, systemic lupus erythematosus and Addison disease, among others [17]. These are autoimmune diseases, that may have affected the melanocytes as “innocent bystanders”. Sunitinib, a tyrosine kinase inhibitor is associated with skin depigmentation [36] and leukotrichia [37] in humans. Vitiligo is also reported in humans with the Vogt-Koyanagi-Harada syndrome [16]. The association of canine vitiligo with the canine uveodermatological syndrome (Vogt-Koyanagi-Harada-like syndrome) is discussed below in this review.

Interestingly, out of 12 canine case reports of vitiligo, two were reported to have a concurrent systemic disease (diabetes mellitus and primary hypoadrenocorticism) [22, 28]. One additional case was linked to the administration of toceranib phosphate [29]. In the study by Mahaffey and colleagues [9], one dog developed hypopigmentation shortly after dexamethasone treatment for demodicosis, another was reported to have irregular estrus cycle and a third dog had a history of pancreatitis. In the dogs of the remaining reports, systemic illnesses were not described.

**Fig. 3:**
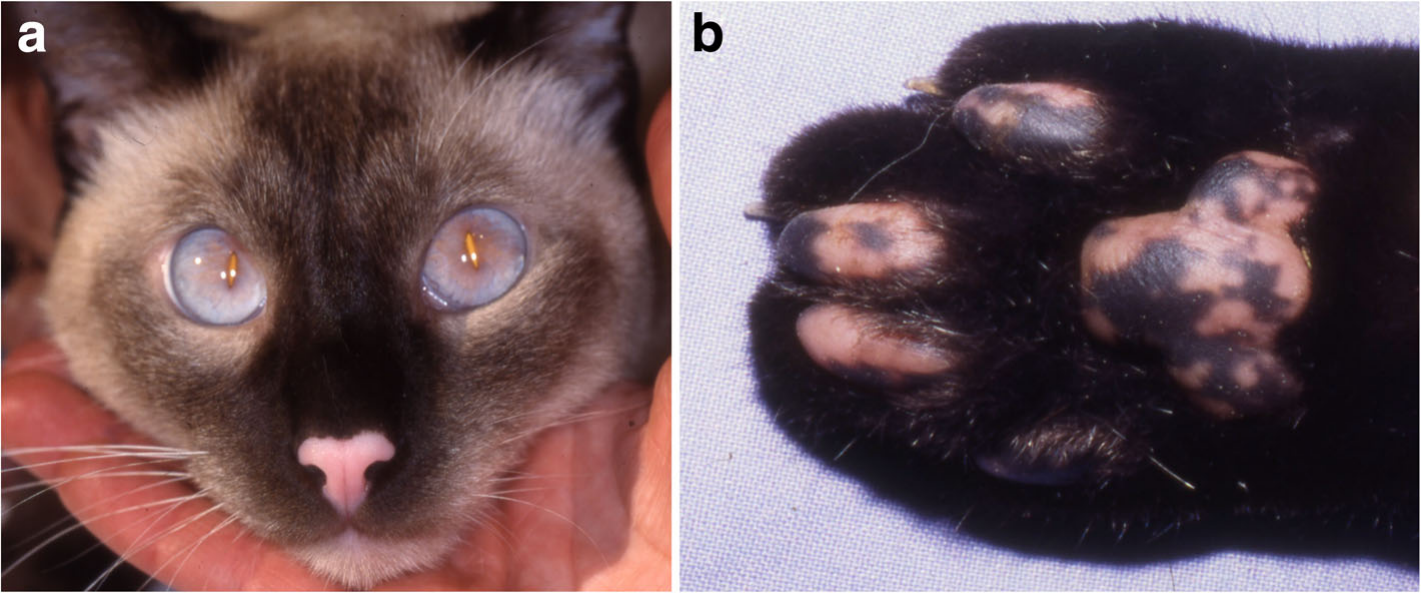
Feline vitiligo. **a** and **b** Siamese cat with vitiligo affecting the nasal planum and footpads (courtesy of Dr. Z. Alhaidari)

In cats, only one report described the locations of the first skin lesions and depigmentation occurred on the nasal planum, periocular area and footpads (Fig. 3) [12]. In three cats, the distribution of lesions were available [11, 12, 30] and the nasal planum/nose was affected in three cats and the footpad(s) in two [11, 12]. Although there are few case reports, this facial-predominant depigmentation in feline vitiligo is similar to what has been reported in dogs. In one cat [12], depigmentation (both leukoderma and leukotrichia) was localized before progressing toward generalization. Systemic illness was not reported in any of these cats.

**Fig. 4:**
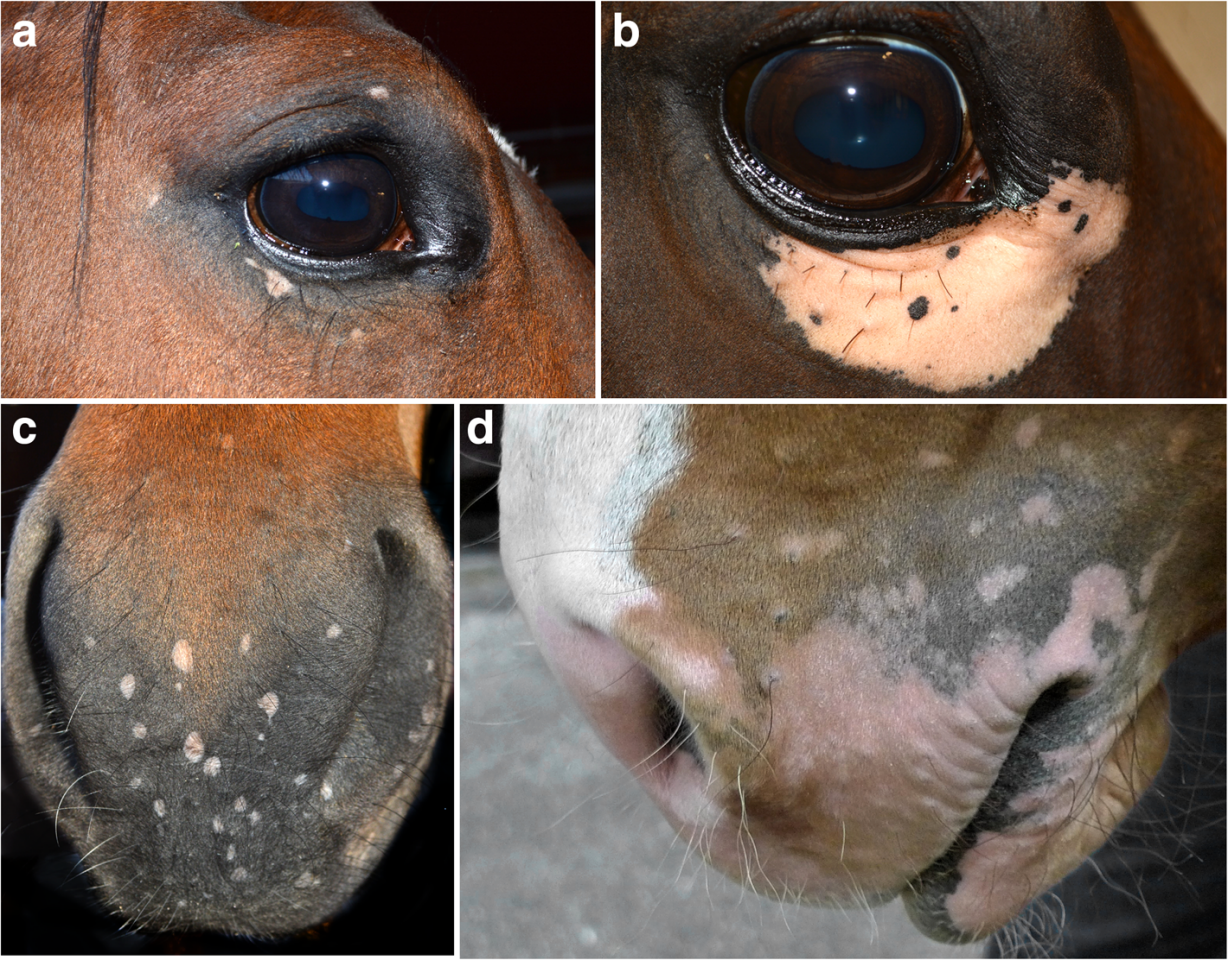
Equine vitiligo. **a** and **c** Quarterhorse with multifocal depigmented macules on the lips, muzzle, face and peri-ocular area (courtesy of Dr. J. Davis). **b** Horse with only a large, unilateral, depigmented patch below the right eye (courtesy of Dr. J. Davis). **d** 1-year-old Swedish warmblood stallion with coalescing leukodermic macules and patches of the lips (courtesy of Dr. K. Bergvall)

Information of the first skin lesion(s) were available for six horses, all of which initially having developed depigmentation on the head/face region [15, 31–33]. The lesion distribution was reported in 11 horses, of which eight (73%) involved the head/face region (Fig. 4). Again, this facial-predominant distribution is similar to that of dogs and cats with vitiligo. However, interestingly, three out of 11 horses (27%) had leukotrichia and leukoderma on the neck, trunk and limbs without involvement of the face or head [15]. Other than vitiligo, an acquired idiopathic leukotrichia, also known as spotted leukotrichia has been reported in horses, especially in thoroughbred, Shire and Arabians [38]. Horses with the so-called “spotted leukotrichia” have multifocal, well-circumscribed, small circular areas of near complete leukotrichia on otherwise normal skin on the neck, trunk and rump [18, 38]. However, to the authors’ knowledge, there are only sparse histologic details on this condition. It is plausible that this spotted leukotrichia is a variant of vitiligo in horses that tends to spare the face and head.

#### Histopathology

**Fig. 5:**
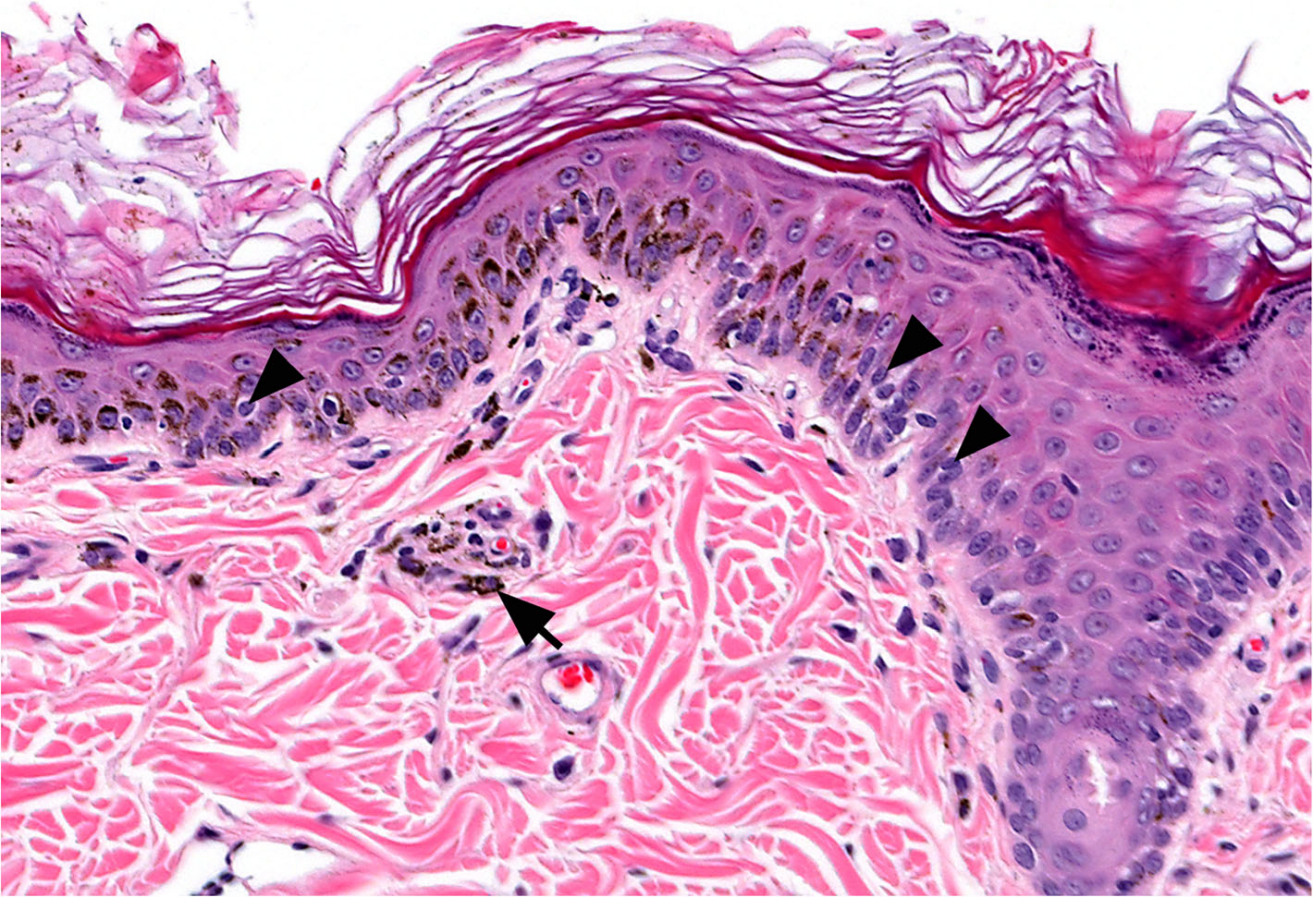
Histopathology of canine vitiligo. In a skin biopsy from the face, lymphocytes infiltrate the basal layer of the epidermis (arrow heads) in very low numbers at the junction of depigmented and non-depigmented epidermis. Melanin containing macrophages are present in low numbers in the superficial dermis (arrow) and are accompanied by a very mild, mononuclear, perivascular, inflammatory cell infiltrate. The epidermal architecture is not significantly altered. Hematoxylin and eosin. 200X

The key histological feature of vitiligo (Fig. 5) in all animal species is the loss of melanocytes from the epidermis and/or hair follicle. The epidermal architecture is normally retained but keratinocytes lack melanosomes (pigment granules) in fully developed areas. Melanosomes are spilled to the superficial dermis (pigmentary incontinence), and sometimes the hair follicle peribulbar area, where they are incorporated in melanophages. Minimal-to-mild numbers of lymphocytes are often present in the basal epidermal layer, especially near the junction of pigmented and nonpigmented epidermis; here lymphocytes are rarely observed adjacent to apoptotic melanocytes (“satellitosis”). Lymphocytes are thought to indicate an active disease state with the cell-mediated destruction of melanocytes. Dermal infiltrates of inflammatory cells, lymphocytes, plasma cells, and histiocytes are minimal or inflammation can be completely absent, then suggesting an inactive disease stage. In the latter situation, histology does not easily differentiate vitiligo from normal white “spots”, but this dilemma can be resolved with the clinical history. Due to the cosmetic nature of the disease, the diagnosis is often made clinically without any need for skin biopsies. If biopsies are to be taken, then multiple samples are ideally collected from the depigmented margins of recently active lesions, especially if erythema were present and indicative of dermal inflammation.

#### Treatment and outcome

In humans, due to the heterogeneity in the designs of clinical trials and often-small numbers of participants, there is no firm clinical recommendation for the treatment of vitiligo [4]. This disease has a devastating psychological impact on the quality of human life, as do psoriasis and atopic dermatitis [16]. This may explain why treatment of widespread, facial or recalcitrant vitiligo in humans can be aggressive.

The Vitiligo Guideline Subcommittee of the European Dermatology Forum outlined the management of NSV based on four levels [3]. The first-line therapy involves narrow band UVB (NB-UVB) radiation whereas treatment escalation involves the use of systemic glucocorticoids (GCs). In non-responding areas, especially those with high cosmetic impact, surgical grafting with the aim to replace the melanocytes with those from a normally pigmented autologous donor site is recommended as the third-line therapy. Finally, in patients with extensive and refractory vitiligo, depigmentation techniques (hydroquinone monobenzyl ether or 4-methoxyphenol) is proposed as the fourth-level treatment. In limited NSV (less than 2–3% of body surface), topical GCs or calcineurin inhibitors and localized NB-UVB radiation are proposed as the first- and second-line therapies, respectively [3]. In humans, acrofacial vitiligo (‘lip-tip’ vitiligo) is resistant to treatment [16], as does a appear the canine disease homologue. Oral and topical JAKinibs, such as tofacitinib, are currently under investigation as mono- or adjuvant therapy for this disease.

In dogs, reports detail a variety of treatments for vitiligo with variable outcomes. These treatments included ammoidin (xanthotoxin) with solar exposure [7], psoralens with ultraviolet light [8], systemic glucocorticoids [9], adrenocorticotrophic hormone (ACTH) injections [9], doxycycline-niacinamide and thyroid supplementation [25], L-phenylalanine [26], vitamin and mineral supplementation [9,25], and diet change [9]. Treatments that resulted in the complete or almost complete repigmentation were psoralens and ultraviolet light [8], as well as ammoidin and solar exposure [7], respectively. In another report [26], L-phenylalanine (a tyrosine precursor) for 6 months resulted in 75% clinical improvement in four dogs. Vitamin and mineral supplementation and diet change resulted in partial repigmentation in some dogs but not others [9]. Adrenocorticotrophic hormone injections over a 3-month period only resulted in a temporary slight improvement in one dog but the depigmentation relapsed when the ACTH injections were stopped [9]. In another dog, a change of environment was suspected to lead to partial repigmentation of the nasal planum and gingiva over several months [9]. Spontaneous remission was reported in two Belgian Tervuerens [9], a litter of seven Labrador retrievers [24], and a Bernese mountain dog [29]. In the latter breed, a spontaneous remission occurred 3 weeks after treatment with toceranib phosphate for mast cell tumour was stopped.

In cats, there are no reports detailing the treatment and outcome of vitiligo. Spontaneous repigmentation, albeit only partial, on the concave and convex surfaces of the pinnae, was reported in one case 40 months after initial onset [12].

Treatment and outcome data for 11 horses were available from three reports [31–33] indicating it took between one to 12 months for significant repigmentation to occur. Complete repigmentation occurred in one horse treated with oral nutritional supplements containing, among others, vitamin A, D, B12 and E [33]. In another report [31], partial repigmentation occurred after supplementation with high levels of chelated copper. A relapse of depigmentation occurred approximately 5 months after the copper supplementation was reduced and then a noticeable clinical improvement occurred when the daily intake of copper was re-increased. It is plausible that the depigmentation in this horse was associated with a copper deficiency and not vitiligo. Nine horses from one case report [32] had complete repigmentation after 1 year of dietary supplementation with carrots (4–5 kg/animal/day). The author suspected that the administration of high levels of thyroprotein-based product might have caused a relative vitamin A deficiency and depigmentation. However, to the authors’ knowledge, there is no evidence that vitamin A deficiency leads to depigmentation in horses but, in humans, several studies have shown that deficiencies of D [39] and B12 [40] vitamins are associated with vitiligo. Therefore, the outcome of these nine horses of this report should be interpreted cautiously. A plausible cause could have been an idiosyncratic reaction to high levels of a thyroprotein-based product, or any of its ingredients, as the cessation of this diet led to spontaneous resolution.

Information on the long-term treatment and outcome of vitiligo in animals is rather sparse, unfortunately, as there are only few published case reports to date.

#### Implications for practice

Vitiligo is a cosmetic issue in animals. Therefore, any treatment for this disease should always be discussed with the owners to avoid interventions of dubious efficacy and those with possible adverse effects. However, vitiligo could have a significant and negative impact for the owners of show animals. In these, the application of topical GCs of high potency (i.e., 0.1% bethamethasone or 0.05% clobetasol) may be attempted on the affected skin, which would only be practical for areas with sparse or no hair; this recommendation is based on the assumption that the pathogenesis of vitiligo in humans and animals is similar and involves an autoimmune mechanism. For areas that have both leukoderma and leukotrichia, the hairs can be clipped to facilitate the penetration of topical GCs into the skin. To avoid localized side-effects of this drug class (e.g., skin atrophy, telangiectasias), the frequency of their application should be tapered to the lowest possible effective one. Topical GCs are still the mainstay of treatment for localized forms of vitiligo in humans [17]. Topical calcineurin inhibitors, such as tacrolimus, offer an alternative to GCs, and, in humans, topical tacrolimus is recommended to be applied twice daily for the first 6 months [4].

Systemic therapy, such as oral or parenteral GCs, is not normally recommended for treatment of vitiligo because the adverse effects outweigh the clinical benefits in animals. If topical therapy were to fail, oral supplementation with L-phenylalanine could be attempted for 6 months, although the clinical remission could be only partial [26]. This supplementation might be tried in addition to the use of topical GCs.

It is important to emphasize to the owners that these treatments, if effective, may prevent the progression of depigmentation but they do not guarantee the partial or full repigmentation.

#### Implications for research

Only two reports [9, 15] of vitiligo in animals had information on their pedigree. In humans, the incidence of vitiligo is higher in those with family history of such disease [19]. It may be worthwhile to perform another study similar to that of Mahaffey and colleagues [9] but at a larger scale (i.e., multicentric study), involving more breeds around different continents to gather more information on the genetic predisposition to vitiligo in animals. This could be followed by a genomic wide-associated study to assess if any variant of vitiligo in some breeds is associated with a single trait. In humans, DDR1, XBP1, NLRP1 and PTPN22 genes have been linked to vitiligo [17] and these genes could be a starting point to investigate if animals with vitiligo are linked to the genes and mutations reported in humans.

Phototherapy with NB-UVB is considered one of the most effective and safest treatment for vitiligo in humans [17], as it results in better repigmentation rates, between 40 and 100% [17]. Interestingly, the dogs that had a complete [8] or an almost complete remission [7] of signs had received a form of phototherapy. Therefore, phototherapy treatment, either alone or in combination with other drugs, warrants further clinical trials in animals.

In humans, the autoimmunity of vitiligo is driven by the interferon-gamma-CXCL10 cytokine signaling pathway [41], which involves the activation of Janus kinases (JAK) 1 and 2. Oral ruxolitinib, a JAK inhibitor with JAK 1 and 2 inhibitory effect, resulted in rapid repigmentation in one man with coexistent vitiligo and alopecia areata [42]. This led to an open-label, phase 2, proof-of-concept clinical trial to investigate the efficacy of topical ruxolitinib 1.5% as a treatment of vitiligo [41]. Should this clinical trial report a noticeable degree of efficacy, it may be worthwhile performing a pilot study with JAK inhibitors in animals with vitiligo. A recent study [43] of topical tofacitinib application in dogs reported anti-inflammatory effect on the skin and therefore, topical JAK inhibitors could potentially be an emerging treatment for various inflammatory diseases in animals. The use of oral JAK inhibitors deserves further investigation, at least in dogs with vitiligo.

Lastly, clinical and histological features of spotted leukotrichia in horses should be gathered and examined to determine if this condition is a variant of vitiligo, or a separate disease condition.

### The uveodermatological syndrome

#### Introduction

The uveodermatological syndrome (UDS) is a canine entity that resembles the VKH disease in humans; in UDS, dogs develop severe bilateral granulomatous posterior or panuveitis with retinal detachments, disk edema and vitritis; these may or may not be accompanied with the tinnitus, hearing loss, vertigo, meningitis, poliosis (patches of hair depigmentation or leukotrichia) and vitiligo seen in the human disease [44]. Herein, we will review the available information published to date on the canine UDS, and where relevant, comparisons with the human homologue (VKH disease) are made. To the authors’ knowledge, UDS has not been reported in cats and horses.

#### Historical perspective

More than a century ago, in 1906, a medical resident from Switzerland by the name of Alfred Vogt published a case report entitled “*Premature whitening of eyelashes and comments about the so-called sudden onset of this change*” [45]. His paper focused mainly on poliosis (patches of white hair) and only briefly described uveitis. Seventeen years later and nearly 6,000 miles away, Einosuke Harada, reported nine cases of “acute diffuse choroiditis” between 1923 and 1926, which later was called Harada’s disease [45]. Twenty-three years later, another Japanese ophthalmologist, Yoshizo Koyanagi wrote a review article that described in detail a disease including severe uveitis, poliosis, alopecia and dysacusis [45].

In 1939, the Vogt-Koyanagi (VK) syndrome was first proposed for the disease reported by Vogt and Koyanagi [45]. Although the disease reported by Harada greatly resembled that of the VK syndrome, it was not until the late 1950s that the term Vogt-Koyanagi-Harada (VKH) syndrome was used [44]. In 2001, the term “disease” was finally selected by the International Committee on Nomenclature of VKH [46], but many papers published thereafter still used the term “syndrome”.

In animals, a disease resembling the VKH syndrome was first reported in 1977 –coincidentally also in Japan [47]. It was not until 1985 that the term “uveodermatological syndrome - UDS” was introduced into the veterinary literature by Romatowski [48]. This author argued that the term “canine VKH syndrome” was inaccurate because the dogs reported did not seem to exhibit any meningeal involvement, and thus did not fit the inclusion criteria of the then human VKH syndrome. It is possible, however, that such neurological signs might have been underdiagnosed. Since then, the terms UDS and VKH-like syndrome have been used interchangeably in the veterinary literature.

#### Etiopathogenesis

The exact etiology and pathogenesis of the VKH disease in humans remain unclear, but the general agreement is that it is an autoimmune disease that targets melanocytes or melanocyte-associated antigens (i.e., tyrosinase and gp100) [49] with an increased susceptibility in populations with certain human leukocyte antigens (HLA) [50]. Indeed, several studies have shown a strong association of HLA-DRB1*0405 and HLA-DQB1*0401 in patients with the VKH syndrome in China, Brazil, Korea and Saudi Arabia [50]. Immunohistochemical studies have shown that 70% of the lesional lymphocytes were T cells [51] and that the choroidal infiltrate was composed predominantly of helper T cells [52]. Other studies [53, 54] showed that, in humans with this syndrome, tyrosinase peptide antigens are the target for autoimmune T cells. Altogether, these data support an autoimmune disease with cell-mediated immunity playing an important role in its pathogenesis.

Interestingly, in some human patients, viral infections (i,e., the Epstein-Barr virus and the cytomegalovirus) have been hypothesized as possible triggering factors of this disease [50, 55]. This could be due to similarities between viral antigens and proteins from pigmented cells (molecular mimicry theory) [50]. The role of antiretinal antibodies (ARAs) in the human VKH syndrome remains the matter of debate because the autoreactivity against retinal proteins seems to differ between acute and chronic disease and these antibodies could be a produced in response to the retinal damage [55]. In addition to this syndrome, antiretinal antibodies have been also detected in other ocular diseases such as cancer-associated retinopathies, toxoplasmosis and age-related macular degeneration [56].

In animals, the UDS likely also has an autoimmune basis. In one study, the dog leukocyte haplotype (DLA)-DQA1*00201 occurred at a higher incidence in American Akitas with UDS compared with normal unaffected dogs of the same breed; there was a significantly higher relative risk and odds ratio for the development of UDS compared to other DLA class II alleles [57]. The injection of peptides derived from tyrosinase-related protein into rats [58, 59] and in two Japanese Akitas [54] resulted in clinical signs resembling those of the human VKH syndrome. Similarly to humans, ARAs were detected in one dog [60] using an ELISA that utilized bovine retinal extract as an antigen source. In that study, the serum from an Akita was positive for ARAs with a titer ≥1:200 (normal reference interval titers was reported to be between 1:25 and 1:50). Interestingly, when the disease was stable, the ARA titer decreased to 1:25 but increased to 1:200 when a relapse occurred. Although, this observation suggests that ARA titers might correlate with disease activity, it is not known if ARAs are the cause or sequela of a previously-active disease.

#### Incidence and prevalence

In several recent review papers, the prevalence of the human VKH disease in North America is reported to be between 1 and 4% of patients [44, 55], but these papers cite an old study published in 1977 that reported VKH to represent 1% of patients affected with uveitis [61]. Therefore, the “true” prevalence of the VKH disease may be much higher now due to better awareness and access to medical services and advancement in diagnostic procedures.

There are no available data to estimate the global or regional incidence or prevalence of the UDS in dogs. However, the canine UDS has a worldwide distribution and has been reported in dogs from Asia [47, 62–64], Europe [65–67], South America [68, 69] and North America [70–72].

#### Signalment

The human VKH disease predominantly affects people in their second-to-fifth decades of life [50], with a higher occurrence in female patients, but children as young as four-years-old also have been reported to be affected [73, 74]; it is more common in individuals with a pigmented skin [44].

In dogs, Akitas, Samoyeds and Siberian Huskies are breeds suspected to be predisposed to the UDS [72], but this syndrome has been reported in other breeds as well. There are 38 articles that can be grouped for the detailed analysis of 166 dogs with the UDS [47, 48, 57, 62–72, 75–98]. Among these cases, 110 (66%) were Akitas, 14 (8%) were Siberian Huskies and five (3%) were Samoyeds; other breeds only made up less than 3% each of the total number of affected dogs reported to date. The age of onset of this disease varied between 7 months and 13 years of age (median: 3 years, mean: 3.6 years) while the female-to-male ratio was 0.6, thus suggesting that males are affected nearly twice as often as female dogs.

#### Clinical signs

The human VKH disease is typically classified into four stages: prodromal, acute, chronic convalescent and chronic recurrent stages. The prodromal stage lasts from several days to a few weeks [44] and it is characterized by flu-like symptoms such as headache, tinnitus, nausea, neck pain and back pain [50]. The acute stage begins with the development of usually bilateral and posterior uveitis [50], and it is during this stage that the blurring of vision appears, and if untreated promptly, visual fields, color vision and central visual acuity can be compromised [44]. The convalescent stage ensues several weeks-to-months after the acute stage, and it consists of signs of depigmentation of the uveal tissue and/or integument [50]. Finally, in the chronic recurrent stage, a mild panuveitis with recurrent episodes of anterior uveitis occurs and this stage is considered the consequence of an inadequate or delayed treatment [50].

Revised diagnostic criteria (RDC) for human VKH have been proposed in 2001 [46] to facilitate the dissemination of knowledge on the VKH disease and to support collaborative research efforts. These RDC divide the diagnosis of the VKH disease into three categories: complete, incomplete and probable. The first two categories are those needed to make a definitive diagnosis, whereas the probable VKH disease category, also referred to as “ocular VKH disease”, needs a continuous monitoring for the clinical signs that would confirm or refute the definitive diagnosis of this disease [50]. Readers are referred to the article published by Read [46] for more information on the clinical findings that determines the classification of human VKH disease into these three categories (Additional file 1: Table S1).

Out of 134 dogs with a spontaneously-occurring UDS in which information on the location of the first sign/lesion (eye versus skin) was available, 114 (85%) developed ophthalmic clinical signs before the onset, or at least the recognition, of skin lesions. Eleven dogs (8%) [64, 72, 75, 90] had cutaneous lesions that preceded ophthalmic signs, whereas, in nine dogs (9%), the ophthalmic and dermatological lesions developed concurrently, or in an undetermined order [72]. The location of the first sign/lesions were not stated in the remaining 34 dogs [57, 58, 66, 67, 86–89, 98]. The median and mean time-lapse between the ocular signs and skin lesions were 12 and 20 weeks, respectively (range: 4 days to 3 years); most dogs (18/21; 86%) that initially had either the eye or skin affected eventually had the two organs eventually bearing lesions within 6 months of each other [48, 64, 65, 67, 69, 75, 76, 78, 79, 82, 83, 86, 90, 92–95, 97]. In one dog, however, bilateral uveitis developed 10 months after the onset of skin lesions [72].

In canine cases where this information was retrievable [47, 48, 62–67, 69–72, 75–98], the most common presenting ocular signs was blindness or poor/decreased vision (38/68; 56%). This is similar to what was reported by Zarfoss where 26/46 dogs (57%) had bilateral blindness at the initial presentation [72]. Other commonly seen ocular signs were uveitis (27/68; 40%) and conjunctivitis or “red eye” (12/68; 18%); it is not known if the dogs with uveitis had concurrent conjunctivitis, and if the conjunctivitis was associated with the UDS or a separate cause.

**Fig. 6:**
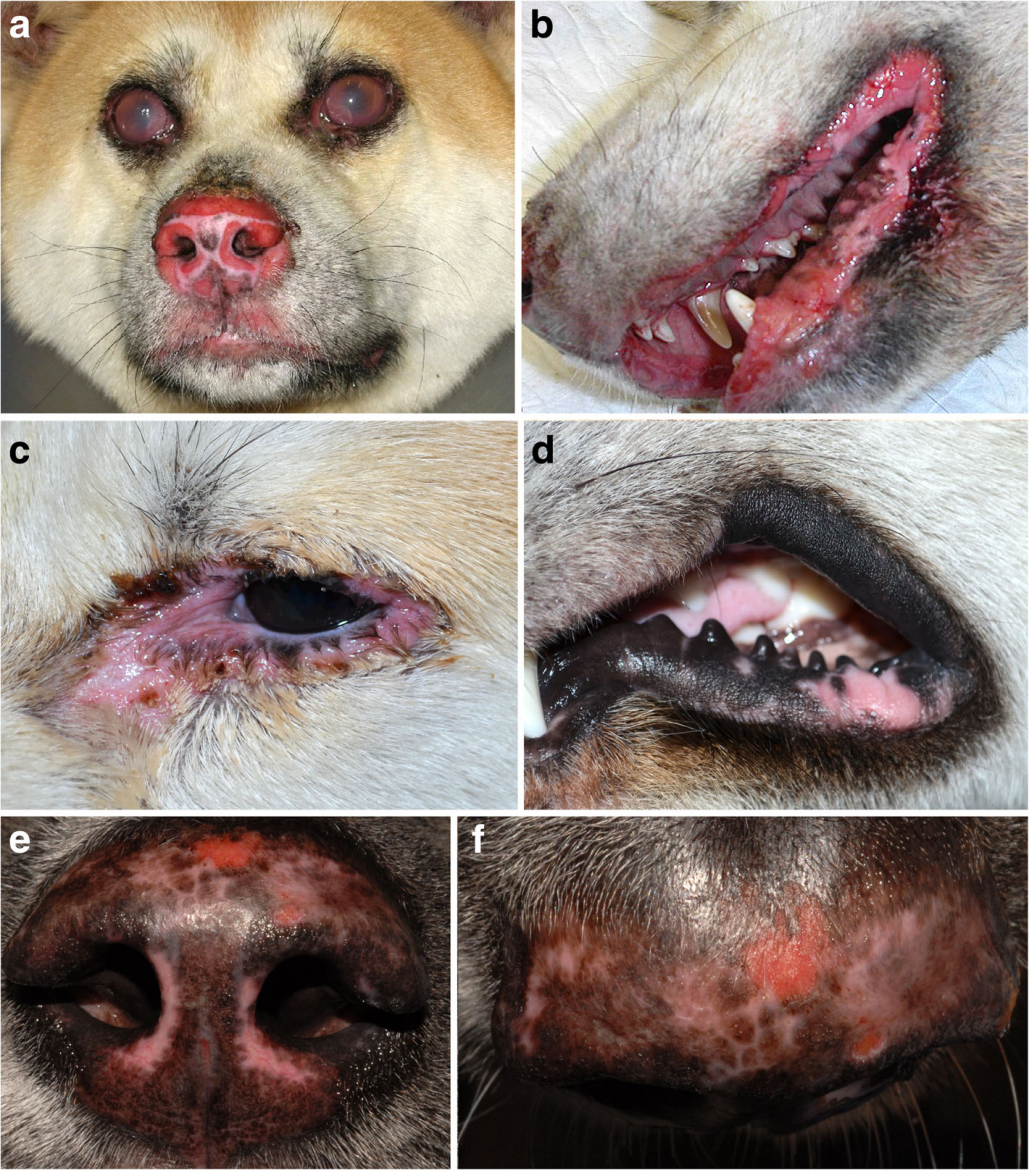
Canine uveodermatological syndrome. **a** chow-chow with bilateral uveitis and severe erosive dermatitis of the nasal planum, and philtrum (**a**), as well as lips and gingiva (**b**) (courtesy of Dr. E. Kuznetsova-Mendoza). **c** 8-year-old Siberian husky with periocular erythema, edema and erosions and, **d** acquired perioral vitiliginous depigmentation; this dog had uveitis 3 months before the cutaneous depigmentation (courtesy of L. Beco). **e-f** 2-year-old Akita inu with uveitis and mottled depigmentation, erythema, focal erosions and loss of the normal nasal planum architecture (courtesy of Dr. F. Banovic, case material NCSU)

The most common skin lesions of canine UDS are leukoderma and/or leukotrichia, followed by erosions-ulcerations, alopecia, crust and erythema (Fig. 6). Other skin lesions reported have been a swelling of the nose [75], pruritus [69], hyperkeratosis of footpads [81] and onychomadesis (loss of claws) [64]. It is interesting to note that the dog reported by Tachikawa developed onychomadesis 1 month after the initial onset of skin depigmentation, and ocular signs only developed 3 months after the first cutaneous lesions. In this dog, it is not known if the onychomadesis was associated with the UDS, was a separate disease (i.e., symmetric lupoid onychitis) that developed concurrently, or was a sequela to UDS due to antigen epitope spreading. Of note and to the authors’ knowledge, onychomadesis has not been reported in humans with the VKH disease.

Among 43 dogs in whom the distribution of skin lesions was reported, all (100%) exhibited lesions on the face or head (Fig. 6) [47, 48, 60, 62–67, 69, 75–79, 81–95, 97, 98]. The most common affected region on the face was the nasal planum (37/43; 86%), followed by the periorbital skin/eyelids (32/43; 74%) and lips (28/43; 65%). Ten dogs (23%) had additional regions affected: the mouth/oral cavity, the footpads and/or the genitalia. Interestingly, all dogs (10/10; 100%) with genital (scrotum and/or prepuce) involvement were males [76, 79, 81, 83–85, 87, 88, 91, 93]. In the oral cavity, the most commonly affected region was the palate (4/10; 40%) [67, 79, 87, 94]. In six dogs (14%), the lesions on the head/face progressed to generalized leukotrichia/leukoderma [69, 77, 86, 88, 91, 97]. Depigmentation of the eyelashes was reported in six dogs (14%) [47, 62, 78, 90].

In all reported cases, the ocular and skin lesions were bilateral and symmetrical except for one dog [90] with iris heterochromia in which uveitis only developed in the right eye with the brown iris; the unaffected left eye had a blue-colored iris.

Concurrent systemic signs were reported in six dogs, shortly before, or at the time when, the diagnosis of UDS was made. The following systemic signs were reported: lethargy [48], a left head tilt with a change in behavior [66], lethargy and cranial nerve II deficit [79], dysacusis [68], left head tilt and reduced appetite [81], and transient pica and depression [82]. In these dogs, the underlying cause of these signs was not determined and therefore, it is not known if they were part of the UDS symptomatology or a concurrent but unrelated finding. One dog was diagnosed with polymyositis 3 years after the onset of uveitis and glaucoma [65].

If the RDC for the VKH disease in humans [46] were applied to all the dogs in this review, most would have been categorized as having either an incomplete or a probable VKH disease. However, it is important to realize that neurological and/or auditory signs in dogs may not be easily observed by the owner or confirmed when presented to the veterinarian, as signs such as meningismus (i.e., the presence of meningeal signs characterized by headache and neck stiffness without actual inflammation of the meninges) and tinnitus (i.e., perception of sound in the absence of external acoustic stimulus) are difficult to confirm or rule-out in dogs and/or these might have resolved by the time of examination. Tinnitus or auto-acoustic emission has been reported in dogs [99] and can be classified as subjective (i.e., noise only heard by the patient) or objective (i.e., noise that can be heard by others) [100]. While the presence of objective tinnitus in dogs very much rely on an astute owner or veterinarian, a subjective tinnitus can neither be proven nor dismissed. Additionally, it is extremely difficult to clinically differentiate meningismus from overt meningitis or meningoenchephalomyelitis in animals [J. Rossmeisl, personal communication]. Nevertheless, there is one case report of a dog with the UDS in whom subclinical involvement of the meninges, based on post-mortem findings, has been documented [84]. Importantly, this dog had not exhibited any clinical signs of meningismus when alive. As a result, the human RDC classification system appears to be of limited use for canine cases. Additionally, it is not clear if the four stages of human VKH disease can be applied to the canine UDS due to an absence of, or a failure to identify prodromal signs. However, most dogs with the UDS exhibit both ocular and dermatological signs, when or soon after being presented to the veterinarian, meaning that they are to be categorized in the convalescent stage.

Three skin lesions listed in the RDC are alopecia, poliosis and vitiligo, and either one needs to be present to fulfill the criteria of human VKH with integumentary involvement [46]. While vitiligo (leukoderma and/or leukotrichia) are the most common skin lesions of the canine UDS, the second most common ones are erosions and ulcerations. One possible reason why skin erosions and ulcers were not included (and therefore assumed to be very rare, if not nonexistent in the human VKH disease) might be due to an earlier diagnosis of this disease in humans, which results in an immediate and aggressive therapy that prevents the development of more advanced skin lesions such as painful erosions or ulcers.

#### Histopathology

**Fig. 7:**
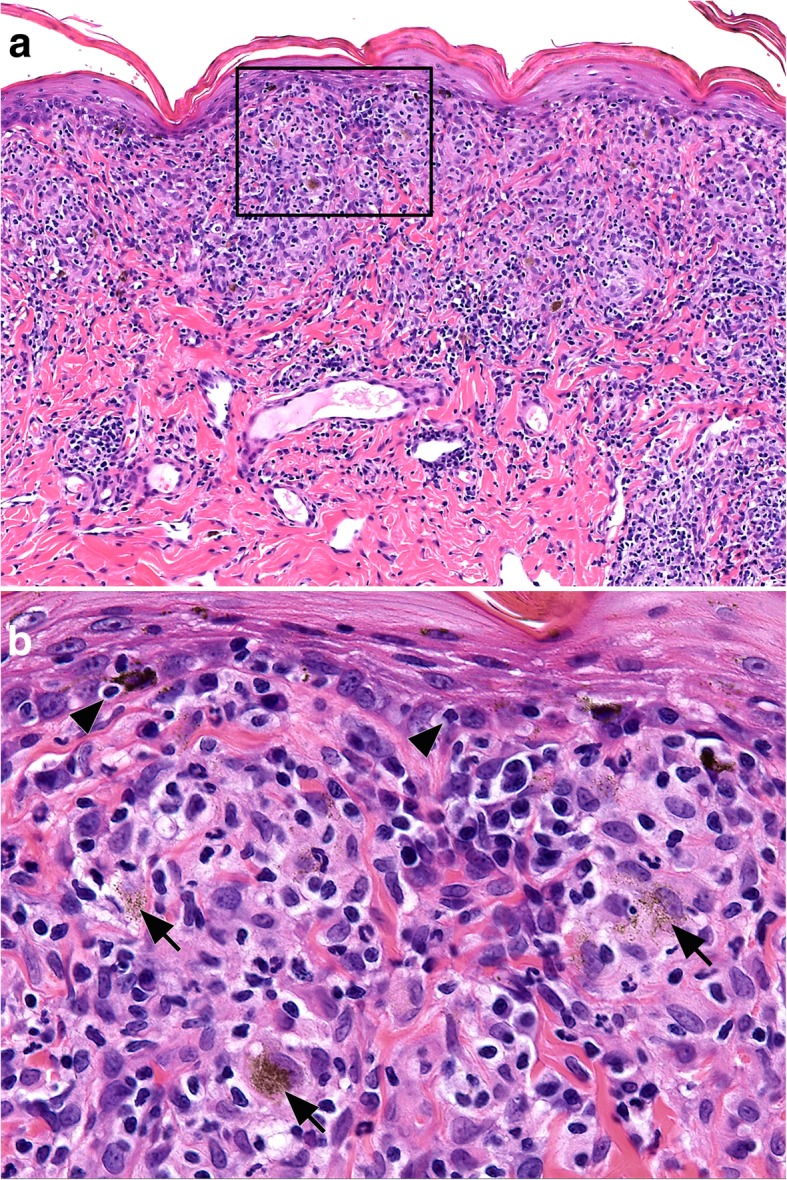
Histopathology of canine uveodermatological syndrome. Skin biopsy from the face of a dog. **a** just below a partially depigmented epidermis, a band-like (lichenoid) dermal infiltrate is dominated by macrophages and lymphocytes with fewer plasma cells and neutrophils. Hematoxylin and eosin. 200X. **b** inset from image (**a**) above: lymphocytes (arrow heads) infiltrate the deep epidermis in low numbers and occasionally appear to surround melanocytes (i.e., “satellitosis”). Melanosomes are spilled into the dermis, where they appear as a fine granular, dust-like, appearance in macrophages (arrows). Hematoxylin and eosin. 400X

Histologically, the canine UDS (Fig. 7a) is characterized by superficial perivascular inflammation that coalesces into a robust lichenoid pattern (i.e., a band-like below the epidermis) that includes macrophages, lymphocytes, plasma cells and a variable number of neutrophils [78]. Macrophages are cited as a prominent feature but might not always be the dominant infiltrating cell type. The exocytosis of lymphocytes into the lower epidermis leads to a blurring of the dermo-epidermal junction, but basal keratinocyte injury and loss—a characteristic interface pattern—is limited or absent. There is partial-to-complete loss of melanocytes and, consequently, of epidermal pigmentation. The apoptosis of melanocytes is expected to occur but is rarely seen. Melanosomes (melanin granules) are spilled to the dermis and are found in melanophages (pigmentary incontinence) were they appear as a finely granular, dusted, cytoplasmic pigment (Fig. 7b). These fine granules have been cited to be a characteristic feature of this disease but the sensitivity and specificity of such microscopic lesion for the diagnosis of the canine UDS has not been proven. Coarsely-clumped pigment can also accompany fine pigment melanophagia. The dermal inflammation pattern is sometimes nodular and periadnexal, but it can be sparse in advanced disease stages. An epidermal hyperplasia is normally seen and can be accompanied by erosions, ulcers, neutrophil transmigration, patchy parakeratosis and/or crust formation. Skin biopsies are indispensible to confirm the diagnosis and should be performed early in suspect cases because of the need to treat early in order to reduce the possibly of blindness. Multiple skin biopsies should be collected from the margins or lesions in areas of recent depigmentation, especially those with active inflammation that exhibit erythema and swelling, which can be subtle.

#### Treatment and outcome

The early and high-dose administration of oral GCs is the mainstay of the VKH disease therapy in humans [44, 50]. Studies have shown that the treatment with high-dose GCs within 2 weeks of disease onset resulted in a shorter duration of steroid use [101], a higher chances of obtaining a complete remission [102] and a shorter duration of disease [103]. The length of systemic GC treatment should also be at least 6 months to reduce the risk of recurrence and severe vision loss [50], but there are no guidelines on the most effective tapering regimen. For patients with a chronic and recurrent disease, or for those who are intolerant to systemic GCs, immunosuppressive agents such as ciclosporin, azathioprine, methotrexate, chlorambucil, mycophenolate mofetil and cyclophosphamide can be used [49]. Whether or not an adjunctive immunosuppressive agents should be employed as a first-line therapy remains a topic of discussion among VKH experts; results of several uncontrolled studies have suggested, however, that an initial multimodal immunosuppressive therapy resulted in a better visual outcome and control of inflammation compared to a monotherapy with GCs alone [50]. Finally, topical GC and cycloplegic agents are indicated to reduce inflammation and pain and to prevent the appearance of synechiae (adhesions). The former can be administered either as ophthalmic drops, intravitreal or subtenon injections [44]. In the human VKH disease, parameters such as the fine visual acuity, the development of cataract, glaucoma or pigmentary changes in the fundus [44, 49] are often used for the assessment on the effectiveness of a treatment protocol. However, there is no agreement on a set of clinical outcomes that would define a patient’s VKH disease as being in clinical remission (CR) or having had a treatment failure.

Similarly, there is no consensus on the definition of CR for the canine UDS, which is largely due to the heterogeneity and lack of standardization of case reports. For the purpose of this review, we will define CR as either one of the following:

1. an improvement or the re-establishment of vision in dogs presented with blindness, or,
2. an absence of development of new signs, or,
3. a lack of progression of lesions (ocular and skin) with resolution of existing ones.

A “failure of therapy” is defined as an inability to control the disease activity (i.e., a continuous development of new ocular signs or skin lesions, a progression/extension of old lesions, or a lack of improvement of ocular signs and/or skin lesions).

Altogether, detailed information about the treatment and outcome of the canine UDS can be inferred from 29 reports [47, 48, 58, 62–65, 67–71, 75, 78, 81, 82, 84–94, 97, 98] including 47 dogs. Another six dogs were either not treated, lost to follow-up or the information on the final outcome was incomplete [66, 77, 79, 83, 89, 95].

Overall, the CR of UDS was obtained in 29/47 dogs (62%). The time-to-CR varied between 2 weeks to 10 months. A spontaneous remission of the UDS has not been reported so far. In dogs for whom follow-up information was available, eight experienced a relapse: clinical signs flared in five dogs when oral GCs were tapered [63, 67, 70, 92] and in three dogs, 3–5 months after treatment was stopped [48, 58, 85].

Treatment regimens have varied widely, and they included the following: GCs (oral, topical ophthalmic and/or subconjunctival), calcineurin inhibitors (ciclosporin or tacrolimus), azathioprine, cyclophosphamide, chlorambucil and mycophenolate mofetil. At the time when a CR was documented, 28/29 (97%) of dogs were treated with oral GCs, of which 18 were concurrently receiving topical ophthalmic, subconjunctival GC or topical ophthalmic calcineurin inhibitors; seven dogs (25%) were concurrently treated with azathioprine. Oral GC monotherapy resulted in a CR of signs in only 3/28 dogs (11%). In most dogs receiving oral GCs, the minimum dosage was 2 mg/kg/day with the dosage slightly lower (1-2 mg/kg/day) in dogs treated concurrently with other immunosuppressants. Interestingly, of 18 dogs (38%) in which a disease CR was not achieved (failure of therapy), oral GCs with or without other immunosuppressant were used as treatment in 15 (83%). The treatment regimen was not stated in one dog that had both eyes enucleated [65].

There is no single treatment protocol that appeared to be associated with the obtention of a more rapid disease CR or a higher treatment success or failure rate. There are 19 case reports [48, 58, 64, 69–71, 75, 78, 81, 82, 85–88, 90–92, 94, 97] where information on the final treatment outcome and time lapsed between the initial onset of signs and the initiation of treatment after the diagnosis of UDS were available – a total of 29 dogs. Of these, 12/20 (60%) and 4/20 dogs (20%) in which CR was achieved, had been treated 1 month and 2–6 months after the onset of clinical signs, respectively. This observation implies that initiating treatment within 1 month after the first clinical signs would result in a better outcome. This is supported by the outcome of 8/9 dogs (89%) in which treatment failed to induce CR that had been treated within 6 months of sign development. This observation is in contrast with the results obtained by Zarfoss et al. [72] who reported that any use of immunosuppressive drugs, duration of signs prior to treatment and high daily doses of GC and azathioprine was not significantly associated with a better prognosis.

#### Implications for practice

Similarly to the human VKH disease, the diagnosis of the canine UDS needs to be made in the shortest time possible, thus allowing for the implementation of an immediate immunosuppressive treatment to prevent disease progression and the development of ocular complications, especially blindness. Because ocular lesions are the most common presenting sign in canine UDS, veterinarians should be extremely vigilant when presented with dogs with non-traumatic or non-infectious signs of conjunctivitis or uveitis, especially in predisposed breeds such as the Akita, Siberian Husky and Samoyed. A complete ophthalmological examination should be performed, and whenever faced with any doubt, the prompt referral to a veterinary ophthalmologist is recommended. On the other hand, dogs presented with only dermatological signs in which histopathology is consistent with a canine UDS, should also undergo a complete ophthalmological examination and continued ophthalmic monitoring even if ocular signs were not reported or if skin lesions were to respond to treatment.

Topical ophthalmic together with systemic GC should be the first-line therapy for the canine UDS, with the dose of oral GC started at 2 mg/kg/day or higher. Other immunosuppressive therapies, such as azathioprine or ciclosporin, should be added into the treatment regimen should GC therapy fail to induce the CR of signs.

#### Implications for research

Specific diagnostic criteria need to be established for the canine UDS. This requires the collaboration of veterinary ophthalmologists, dermatologists and neurologists. The presence or absence of neurological and/or auditory abnormalities in the canine UDS should be investigated further via ancillary test such spinal taps to determine if pleocytosis of the cerebrospinal fluid is present in dogs with this disease. Likewise, diagnostic tests that are more sensitive and can reliably detect tinnitus in dogs should be investigated. The ability to detect prodromal signs (if present) in dogs with the UDS would likely result in a better prognosis because treatment could be started sooner.

A validated scoring system or index for treatment response would be of value, as it would allow for a standardization of the reporting of treatment outcomes among publications, thus allowing for a better assessment and comparison of treatment efficacy. This, in turn, would facilitate the establishment of guidelines for the treatment and management of the canine UDS. With this, the usage of biologic agents with minimal adverse effects could then be explored as the future treatment for canine UDS.

## Conclusions

Autoimmune diseases targeting melanocytes can manifest with a wide range of clinical signs. It remains a mystery as to why “attacks” on the same pigmented cells, the melanocytes, results in just depigmentation of the skin in some dogs or leads to a “catastrophic” effect on the eyes and/or skin in others. Until the exact etiology is known, the treatment of canine vitiligo should take into consideration the efficacy (or lack of) of a particular therapy and weigh the adverse effects of treatment for this mostly cosmetic disease. On the other hand, the prompt implementation of an aggressive immunosuppression cannot be overemphasized for the treatment of canine UDS to prevent blindness in affected patients.

## Additional file

Additional file 1: **Table S1** Diagnostic criteria for Vogt-Koyanagi-Harada disease.* (XLSX 13 kb)

## Data Availability

This article being a review of published information, data sharing is not applicable as no datasets were generated or analysed.

## Abbreviations

ACTH: Adrenocorticotropic hormone
ARAs: Antiretinal antibodies
CR: Clinical remission
GC: Glucocorticoid
HLA: Human leukocyte antigen
JAK: Janus kinase
NB-UVB: Narrow band ultraviolet B
NSV: Non-segmental vitiligo
RDC: Revised diagnostic criteria
SV: Segmental vitiligo
UDS: Uveodermatological syndrome
VK: Vogt-Koyanagi
VKH: Vogt-Koyanagi-Harada
